# Molecular, Viral and Clinical Features of Alcohol- and Non-Alcohol-Induced Liver Injury

**DOI:** 10.3390/cimb44030087

**Published:** 2022-03-16

**Authors:** Manuela G. Neuman, Helmut K. Seitz, Rolf Teschke, Stephen Malnick, Kamisha L. Johnson-Davis, Lawrence B. Cohen, Anit German, Nicolas Hohmann, Bernhardo Moreira, George Moussa, Mihai Opris

**Affiliations:** 1 In Vitro Drug Safety and Biotechnology and the Department of Pharmacology and Toxicology, Temerity Faculty of Medicine, University of Toronto, Toronto, ON M5G 1L5, Canada; george.moses@gmail.com (G.M.); m_neuman@rogers.com (M.O.); 2 Centre of Liver and Alcohol Diseases, Ethianum Clinic and Department of Clinical Pharmacology and Pharmacoepidemiology, Faculty of Medicine, University of Heidelberg, 69115 Heidelberg, Germany; helmut_karl.seitz@urz.uni-heidelberg.de (H.K.S.); nicolas.hohmann@med.uni-heidelberg.de (N.H.); bernardomaikita@gmail.com (B.M.); 3 Department of Internal Medicine II, Division of Gastroenterology and Hepatology, Klinikum Hanau, Hanau, Academic Teaching Hospital of the Medical Faculty, Goethe University Frankfurt/Main, 60323 Frankfurt, Germany; rolf.teschke@gmx.de; 4 Department of Internal Medicine C. Kaplan Medical Center, Faculty of Medicine, Hebrew University of Jerusalem, Rehovot 76100, Israel; steve@stevemalnickmd.com (S.M.); anit.german@clalit.il (A.G.); 5 Department of Pathology, University of Utah Health Sciences Centre and Division of Toxicology, ARUP Institute for Clinical and Experimental Pathology, Salt Lake City, UT 84115, USA; kamisha.johnson-davis@aruplab.com; 6 Division of Gastroenterology, Sunnybrook Health Sciences Centre and Department of Medicine, Temerity Faculty of Medicine, University of Toronto, Toronto, ON M4N 3N5, Canada; lawrence.cohen@sunnybrook.ca; 7 Family Medicine Clinic CAR, 010362 Bucharest, Romania

**Keywords:** alcoholic liver disease, apoptosis, cellular toxicity, cytokines, cytochrome P450, inflammation, fibrosis, microsomal ethanol oxidizing system, reactive oxygen species

## Abstract

Hepatic cells are sensitive to internal and external signals. Ethanol is one of the oldest and most widely used drugs in the world. The focus on the mechanistic engine of the alcohol-induced injury has been in the liver, which is responsible for the pathways of alcohol metabolism. Ethanol undergoes a phase I type of reaction, mainly catalyzed by the cytoplasmic enzyme, alcohol dehydrogenase (ADH), and by the microsomal ethanol-oxidizing system (MEOS). Reactive oxygen species (ROS) generated by cytochrome (CYP) 2E1 activity and MEOS contribute to ethanol-induced toxicity. We aimed to: (1) Describe the cellular, pathophysiological and clinical effects of alcohol misuse on the liver; (2) Select the biomarkers and analytical methods utilized by the clinical laboratory to assess alcohol exposure; (3) Provide therapeutic ideas to prevent/reduce alcohol-induced liver injury; (4) Provide up-to-date knowledge regarding the Corona virus and its affect on the liver; (5) Link rare diseases with alcohol consumption. The current review contributes to risk identification of patients with alcoholic, as well as non-alcoholic, liver disease and metabolic syndrome. Additional prevalence of ethnic, genetic, and viral vulnerabilities are presented.

## 1. Microsomal Ethanol Oxidizing System Due to Cytochrome (CYP) 2E1 Rather than Catalase or ADH

### 1.1. The Discovery of MEOS

Charles Saul Lieber, M.D., Professor of Medicine and Pathology, was a pioneer in the field of alcohol-related liver disease, including the hepatic alcohol metabolism. His discovery of the MEOS, which functions in the absence of ADH and catalase but requires cytochrome P450 2E1, changed the clinical concept of ethanol metabolism. 

The MEOS is a unique liver enzyme system which metabolizes ethanol and activates alcohol and drug interactions leading to liver injury [1,2,3]. The discovery and detailed characterization of MEOS was the result of his observation that prolonged alcohol use in humans and animals and caused a proliferation of the smooth endoplasmic reticulum (SER) of the hepatocytes. Both catalase and ADH are known as contaminants of microsomal fractions at variable degrees. Lieber and his associates demonstrated the importance of MEOS [4,5,6] to the scientific world. 

### 1.2. Initial Studies on MEOS Isolation

**Figure 1:**
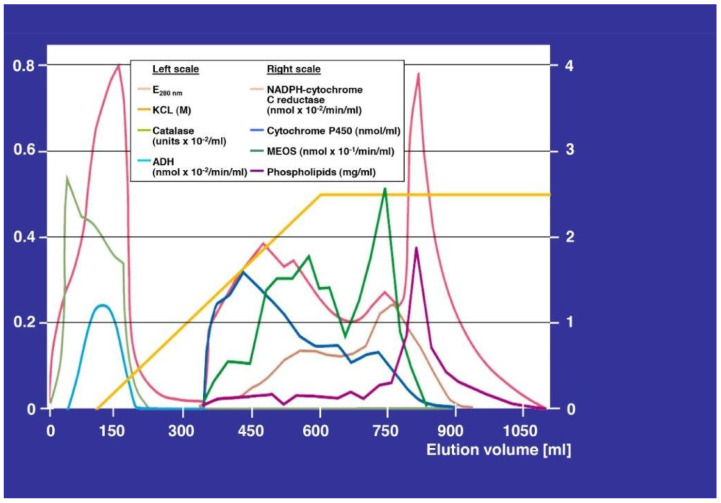
The elution pattern with separation of the MEOS from catalase and ADH activities. Using ion exchange DEAE column chromatography and a continuous KCl grading starting with 0 and increasing up to 0.5 M KCl (bright yellow), eluates started with the void volume recovered up to around 220 mL. The highest peak represents the protein curve (light red) assessed as E_280nm__._ The left-sided peak below represents the catalase peak. ADH is the lowest peak. Starting with an elution volume at 330 mL, the microsomal components appears. Cytochrome P450 (blue) can be seen around 440 mL that decreases in the further course up to 900 mL, the second peak at 500 mL represents E_280nm_ (light red), followed by a third peak at 580 mL with two shoulders, and by a fourth peak at 740 mL representing MEOS (green). At 750 mL, the reductase peak emerges (light violet), followed by the phospholipids peak (dark violet) at 790 mL elution volume. Modified from the original figure published in a previous report [8].

The MEOS project was a joint action at the Bronx VA Hospital-affiliated Mount Sinai Hospital in Manhattan. In the same period of time, Lu and Coon [7] were working at solubilization of MEOS. MEOS could be solubilized from the liver using ultrasonication. The metabolically active MEOS was separate from ADH and catalase. These results confirmed MEOS as a separate enzyme system. The results shown in Figure 1 have been published previously [8].

### 1.3. Components of MEOS: CYP, Reductase and Phospholipids 

The analysis of the elution pattern of MEOS is shown in Figure 1. MEOS activity was found only in fractions that contained CYP, reductase, and phospholipids. However, the MEOS activity did not parallel CYP activity. In eluates containing lower amounts of CYP and phospholipids, the MEOS and reductase activities presented the highest peaks. In addition, MEOS activity was not found in eluates containing both, the reductase and phospholipids, or phospholipids alone. The turnover number of MEOS activity in relation to the CYP may reflect different CYP isoforms, which exhibit variable affinities for ethanol. The methods to characterize CYP isoform were not available at time of publication in 1972 [8].

MEOS activity was restricted to fractions free of ADH. The microsomal components CYP, nicotinamide adenine dinucleotide phosphate (NADPH) reductase, and phospholipids have catalytic and peroxidic activities.

In subsequent studies with usual intact microsomes or the solubilized, isolated and purified MEOS fraction, the microsomal system was not only active with ethanol as a substrate, but also oxidized higher aliphatic alcohols such as propanol, butanol, and pentanol. None of these are substrates for the peroxidatic activity of catalase [9,10,11,12]. These results confirmed that microsomes contain an enzyme system that is active in the absence of catalase and ADH. 

### 1.4. MEOS Oxidized Higher Aliphatic Alcohols

The purified MEOS oxidized also higher aliphatic alcohols. The oxidation was dependent on NADPH and molecular oxygen. It had a Michaelis Menten constant of 7.2 mM for ethanol. The process is active at intermediate and higher alcohol concentrations. The same concentration of alcohol can be found among individuals consuming alcoholic beverages in higher amounts, a pH in the physiological range of 6.9–7.5 and that was insensitive to ADH and catalase inhibitors [13]. 

**Figure 2:**
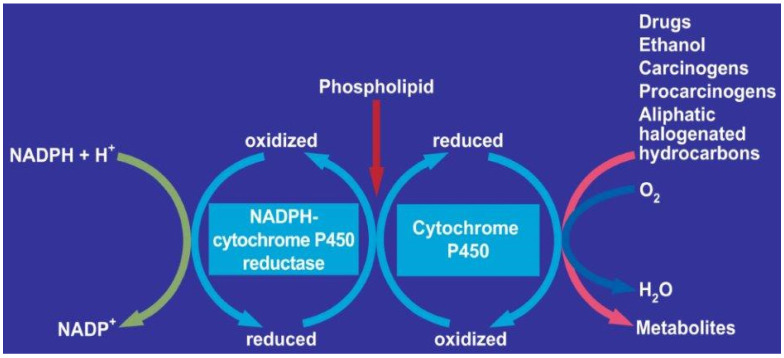
MEOS and other exogenous substrates. This schematic sequence of events at the microsomal level was expanded and modified from a figure previously published in an open-access journal [13].

Cytochrome P450, NADPH, CYP reductase, phospholipids, and MEOS share the same metabolic pathway. Other hepatic microsomal enzymes, metabolizing drugs, carcinogens, procarcinogens, or aliphatic halogenated hydrocarbons (AHH) such as carbon tetrachloride share the same pathway (Figure 2) [13].

In order to catalyse ethanol oxidation, ADH needs cofactor nicotinamide adenine dinucleotide (NAD^+^), which is reduced to NADH and acetaldehyde [14]. Because of its electrophilic nature, acetaldehyde is able to covalently bind to proteins, lipids and nucleic acids and form acetaldehyde adducts that alter intracellular homeostasis [15]. Cellular proteins become highly susceptible to proteolysis, changes in their electric charge, as well as to enzyme inactivation. 

Inside mitochondria, acetaldehyde is oxidized to acetate by enzyme ALDH_2_. This reaction generates acetate and even more NADH [16,17]. As a result, cellular NAD^+^/NADH ratio decreases, this having a strong impact on the inhibition of fatty acid beta-oxidation causing tri-acyl-glycerols to accumulate inside hepatocytes [18]. Besides its important role in the development of liver steatosis, the generation of NADH has other significant effects on citric acid cycle, gluconeogenesis and glycolysis. Inside the mitochondrion, NADH is re-oxidized to NAD+ by the electron transfer chain. While electrons are transferred towards oxygen in the inner mitochondrial membrane, a high amount of reactive oxygen species (ROS), such as the hydroxyl radical (OH), superoxide anion (O_2_) and hydrogen peroxide (H_2_O_2_), is formed. Regarding nucleic acids, both nuclear and mitochondrial deoxyribonucleic acid (DNA) are vulnerable in front of ROS. 

### 1.5. Cellular Proteins

Any impairment in the genetic code will affect the rightful synthesis of cellular proteins. Several studies have shown that 8-oxo-guanine is the main marker of alcohol-induced DNA damage. Its level has been measured in rat’s liver after chronic alcohol administration [13]. CYP2E1 is located mostly in the endoplasmic reticulum. In the presence of molecular oxygen, CYP2E1 oxidates ethanol to acetaldehyde and transforms reduced NAD phosphate (NADPH) to NADP ^+^ water. Being an inducible enzyme, CYP2E1 is able to increase its levels, especially during chronic alcohol consumption [14,15]. Whenever CYP21E1 exceeds its normal level, molecular oxygen becomes an important substrate for the enzyme, displaying a high amount of ROS such as superoxide anion (O_2_), hydrogen peroxide (H_2_O_2_), hydroxyl radical and hydroxyethyl radical (HER) [16,17]. Out of the microsomal cytochrome P-450 enzymes, CYP2E1 is the most susceptible one, when it comes to producing ROS because of its weak connection with the CYP redox cycle and with NADPH-cytochrome P450 reductase [16,17,18]. Two days are needed in order to achieve results from the isolation procedure. On the first day, microsomes are solubilized and submitted to column chromatography in the cold room. In the afternoon of the same day, the eluates are obtained. Overnight dialyses, in the cold room, is required to separate the eluates from toxic deoxycholate.

Later studies from Dr. Lieber’s group showed that MEOS activity can be reconstituted with CYP 2E1, the reductase and phospholipids [16]. It became clear that CYP 2E1 is the most active isoform. In addition, CYP 2E1 is responsible for the increase of MEOS activity after prolonged alcohol consumption. These conditions explain the enhanced metabolic rate of ethanol disappearance observed in individuals with an increased alcohol use over a longer time. Point by point, all of our initial results of MEOS isolation were independently confirmed by Damgaard [19].

### 1.6. Role of CYP 2E1 for Ethanol in MEOS and Other Exogenous Substrates

**Figure 3:**
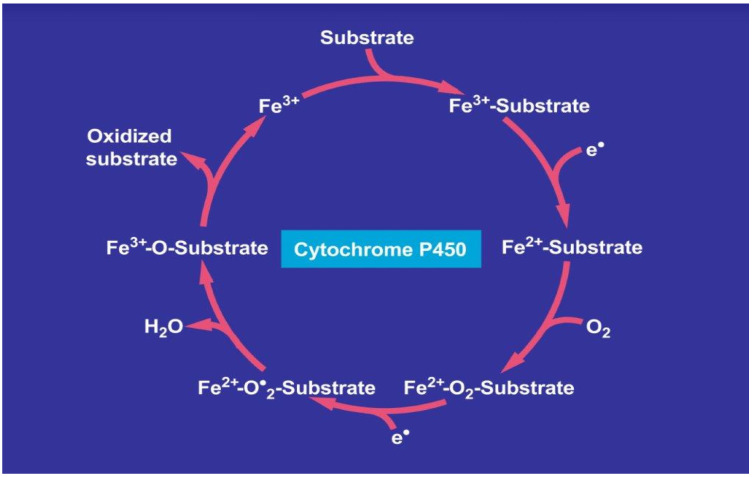
Catalytic CYP 2E1 cycle of MEOS. CYP reaction with Fe^2+^ is generated; after splitting off the oxidized substrate (ethanol), Fe^2+^ is oxidized again. CYP became then again free and is oxidized again.

Recognizing that CYPs, and especially its isoform CYP 2E1, are part of MEOS, was an important achievement, elucidating possible mechanistic steps in MEOS activity. Ethanol may be oxidized via the CYP 2E1 catalytic cycle. A tentative step-by-step approach is presented (Figure 3) [13].

In analogy to other exogenous compounds, ethanol enters the catalytic cytochrome P450 cycle as substrate as shown on top of the cycle. After several steps, the metabolized ethanol leaves the cycle, providing acetaldehyde as oxidized substrate**.** CYP stands for its various isoforms. The term “P450” was used to describe a “pigment” with an absorption maximum at 450 nm with the ferrous-carbon monoxide complex of CYP in rat liver microsomes. The figure was retrieved from an open-access report [13]. 

**Figure 4:**
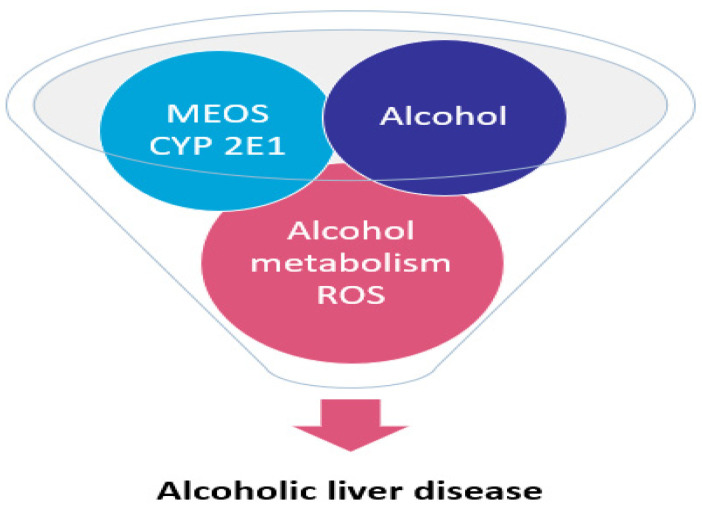
Co-triggering role of MEOS, CYP 2E1, and ROS in alcoholic liver disease.

More specifically, the first electron is provided to CYP by NADPH + H^+^ via the NADPH CYP reductase. The reduced form of CYP with Fe^2+^ is generated, which finally becomes oxidized again after splitting off the oxidized substrate. CYP is then again free for the next substrate to be oxidized (Figure 3) [13]. Through the introduction of molecular oxygen, a multi-compound reactive complex emerges, facilitated by inclusion of another electron that commonly is provided through the NADPH-CYP reductase or NADPH independent reductase. Under normal conditions, CYP dependent enzymatic event proceeds smoothly, but occasionally, ROS is generated from incomplete split of oxygen leading to liver injury [17,18,19,20,21,22]. In Figure 4, there is a graphic representation of liver injury due to the MEOS, CYP 2E1 activation and ROS.

This figure was taken from an open-access publication [22]. 

Among the potentially toxic intermediates generated comprised as ROS, some are listed (Table 1).

The molecular mechanisms whereby ethanol is metabolized to acetaldehyde via MEOS and CYP 2E1 brings also the important role of hydroxyl radicals. Studies with hydroxyl radical inhibitors support this observation. In addition, ethanol is a potent hydroxyl radical scavenger, whereas a lack of inhibition by dismutase would negate the involvement of superoxide radicals.

### 1.7. CYPs Inducers of Other Substances

Both acetaminophen and ethanol are inducers of CYP 2E1. This explains the hepatotoxic effect of acetaminophen in persons taking a normal dose of acetaminophen after being intoxicated with alcohol. Table 2 summarized substrates of CYP 2E1.

Alcohol research was enriched by the work of Charles S. Lieber, who discovered MEOS as a new liver enzyme metabolizing ethanol, without requiring ADH or catalase.

### 1.8. Various Aspects

Among the major research studies are acetaldehyde in the liver [23], hepatic CYPs isoforms, genetic polymorphisms [24,25,26], mitochondrial CYP 2E1 [27], biochemical studies [28,29,30,31,32,33,34,35,36], inhibition of CYP 2E1 with chlormethiazole [37], and the gut microbiome [38,39]. Forsyth et al. 2014 [40] showed the effects of alcohol-induced gut leakiness. The toxins from the gastrointestinal system arriving to the liver induce steatosis, and apoptosis. Also, the studies by Méndez-Sánchez et al., 2020 [41], described the link of alcohol leading to liver cancer. 

### 1.9. Alcoholic Liver Disease: The CYP2E1

#### 1.9.1. Hydroxy-Nonenal Which Binds to DNA and Etheno-DNA-Adducts

The metabolic consequences of this CYP2E1 induction are increased ethanol metabolism, interaction with the metabolism of drugs, xenobiotics and carcinogens leading to increased toxicity and carcinogenesis, an enhanced degradation of retinol and retinoic acid, and most importantly, the generation of ROS, which contribute to liver damage. CYP2E1 activity correlates with the generation of lipid-peroxidation products (4-hydroxy-nonenal), which bind to DNA and form carcinogenic etheno-DNA-adducts. 

In agreement with this concept, CYP2E1 knock-out mice showed less severe ALD and CYP2E1 over-expressing mice a significant deterioration of ALD following ethanol administration [29,42]. 

The discovery of Clomethiazole (CMZ) as a strong non-competitive CYP2E1 inhibitor with a Ki of 12 µM was a step forward to prove the involvement of CYP2E1 in ALD. 

CYP2E1 is involved in retinoic acid (RA) metabolism [43,44]. Rats, which were chronically fed with alcohol, showed a significantly lower RA concentration in the liver, related to an increased CYP2E1 activity. 

Inhibition of CYP2E1 cannot only reduce oxidative stress, but also restore normal retinoid signals and functions, which, in turn, might reduce the risk of cancer. Administering pure ß-carotene, retinol or RA is dangerous, since these substances increasingly develop into toxic metabolites when CYP2E1 is induced [45,46]. Thus, an administration of vitamin A or ß-carotene together with alcohol leads to increased hepatocellular damage [47]. The hepatic RA values completely normalized after inhibition of CYP2E1 by CMZ as part of the diet [48]. These results show that CMZ is able to normalize hepatic retinol and RA concentrations as well as retinoid signals by inhibiting their degradation through CYP2E1. CYP2E1 inhibition not only normalizes hepatic RA concentrations, but also other functions such as cell proliferation and cell cycle behavior [49]. 

#### 1.9.2. The Randomized Controlled Clinical Trial with CMZ

We performed a randomized controlled clinical trial with CMZ to inhibit CYP2E1 vs. clorazepate (CZP), without such potential, in ALD patients admitted to the hospital for alcohol detoxification therapy [50]. Sixty patients were included in the study. Immediately after enrolment, we performed a chlorzoxazone test to determine CYP2E1 activity. Thereafter, patients were randomized to receive either CMZ or CZP and these drugs were dosed according to clinical withdrawal symptoms. CMZ was started with 6 to 10 times 10 mL/d (315 mg) and CZP with 4 to 6 times 25 mg/d. Following enrolment, determination of serum transaminase activities was repeated on days 1–7 and at discharge. After the first chlorzoxazone test immediately after enrolment, the test was repeated 24 h later and at discharge. 

Already after 4 days of treatment, AST and ALT activities were significantly lower in CMZ patients compared to patients on CZP. This difference became more significant at discharge. In addition, both CMZ and CZP decreased hepatic fat significantly (*p* < 0.0001 vs. baseline). This randomized controlled clinical trial demonstrates, for the first time in humans, that CMZ, with its CYP2E1 inhibiting effect, accelerates recovery from ALD at an early stage, as confirmed by a more rapid improvement of serum transaminase activity and as compared to treatment with benzodiazepines [50,51].

In conclusion, inhibiting CYP2E1 activity is a possible therapeutic way to reduce the alcohol-induced injury to the liver. 

## 2. Biochemical Laboratory Testing for Biomarkers of Alcohol Exposure

### 2.1. The Need for Biomarkers of Alcohol Exposure

Alcohol is a major contributor to global disease and a leading cause of preventable death, causing approximately 88,000 deaths annually in the United States alone. Alcohol use disorder (AUD) is one of the most common psychiatric disorders, with nearly one-third of U.S. adults experiencing alcohol AUD at some point during their lives. Alcohol use disorder also has economic consequences, costing the United States at least $249 billion annually. Current pharmaceutical and behavioral treatments may assist patients in reducing alcohol use or facilitating alcohol abstinence.

In the United States, according to the National Institute on Alcohol Abuse and Alcoholism, alcohol users are classified as ‘moderate’ if they consume 1–2 drinks/day; ‘binge drinker’ if they consume 4–5 drinks within a short time period, and ‘heavy alcohol use,’ if they binge drink for 5 or more days [52]. Out of the 51% of the adult population that uses alcohol, 48% self-identified as a binge drinker and 12% of the alcohol user group identified as heavy drinkers [52]. Consequently, there is a clinical need to test for alcohol exposure. 

Testing for alcohol exposure is imperative to support the emergency department to manage the health of patients with alcohol intoxication, it is used to test for driving under the influence, to assess adherence for pain management programs, drug and alcohol abstinence programs, and prequalification screening for organ transplantation surgery. It is also important for child welfare cases in order to make informed decisions regarding family support services, family reunification, child removal, or termination of parental rights. Alcohol abstinence is an important eligibility criteria for patients waiting on the organ transplant list; therefore, long-term alcohol biomarkers are needed to monitor alcohol use in patients before and after transplantation, in order to intervene or refer patients for alcohol treatment programs as needed. 

Sexually dimorphic effects of alcohol exposure throughout life have been documented. The difference between sexes in prevalence of AUD and binge drinking has narrowed. Recent evidence adds to historical data regarding the influence of sex steroids on alcohol drinking and the interaction with stress-related steroids [53]. 

In the United States, about 1 in 9 pregnant women consume alcohol during pregnancy. Among these pregnant women, about one-third engaged in binge drinking, with an average of 4 to 5 binge-drinking episodes in the past 30 days. Alcohol use during pregnancy is a concern because alcohol in the mother’s blood passes to the baby through the placenta and umbilical cord. In addition, amniotic fluid can serve as a reservoir to prolong fetal exposure to alcohol. The metabolite of alcohol, acetaldehyde, is toxic and can disrupt DNA and protein synthesis and cell growth [54].

### 2.2. Biomarkers

The laboratory can offer testing for alcohol biomarkers and there are several specimen types that can be used to assess alcohol exposure. Urinary markers for ethanol use consist of ethanol glucuronide (EtG) and ethanol sulfate (EtS), which have shown to provide a window of detection of alcohol consumption up to 1 week after use and are not impacted by the presence of liver disease [54].

Ethanol < 0.1% is metabolized by UDP-Glucuronosyl-transferase to form EtG. Sulfotransferase metabolizes < 0.1% of ethanol forming EtS. Whole-blood specimens are often evaluated for forensic testing to determine the blood–alcohol concentration, which correlates to the degree of impairment. Phosphatidyl-ethanol (PEth) is a direct ethanol marker that detects chronic heavy drinking in blood with high specificity and is not affected by liver disease. Phosphatidyl-ethanol is an abnormal phospholipid formed in the red blood cell (RBC) membrane in the presence of ethanol, catalyzed by the enzyme phospholipase D [55].

The half-life of PEth is 3–8 days and it has a window of detection of 28 days after ethanol use [55]. This marker can be analyzed by mass spectrometry and may be beneficial for detecting ethanol use during pregnancy or when a longer window of detection is needed to identify ethanol consumption. Fatty acid ethyl ester is a biomarker that can be used to assess neonatal alcohol exposure and has a window of detection of up to 20 weeks prior to birth and umbilical cord tissue is utilized to detect EtG within the last trimester of pregnancy [56].

Breath and saliva testing are specimens used by the department of transportation for roadside testing and saliva can be used for workplace drug screening. Analysis of breath is commonly used by law enforcement to determine recent alcohol use associated with driving. This involves the oxidation of expired ethanol to acetic acid and water in the flow cell of the analyzer. An electrical current is measured in proportion to the amount of acetic acid produced and an alcohol concentration is extrapolated. Ethanol is also commonly measured in blood by extracting vaporized ethanol from the headspace of a sealed vessel containing the blood sample or by direct injection without headspace. 

This vapor (or prepared sample) is injected onto a gas chromatograph and a detector response (typically flame ionization) is measured proportional to the concentration of ethanol in the sample [57]. The method is also useful in distinguishing specific alcohols in the system (e.g., ethanol, methanol, isopropanol, etc.). A major disadvantage to measuring ethanol directly is its short half-life (2–14 h). In clinical settings, plasma and serum samples are typically used to quantify ethanol or to evaluate liver enzymes. 

Carbohydrate deficient transferrin (CDT) is a serum marker for heavy, long-term alcohol use. CDT refers to isoforms that are deficient in sialic acid, such as glycoforms (asialo and disialo-Transferase). CDT is formed in individuals with heavy alcohol consumption of about 60–80 g/day for at least two weeks [58]. 

The mechanisms responsible for the increase of CDT are not clearly understood, but it is hypothesized that heavy alcohol use may decrease the activity of enzymes responsible for the incorporation of sialic acid in transferrin molecules [59]. CDT is reported as a percent of total transferrin and a positive cutoff is ≥1.7%. It is important to note that CDT testing cannot be used in individuals suspected of having congenital glycosylation disorders and that pregnant women, during the 3rd trimester, will have elevated serum disialo-transferrin [60].

The analytical methods used to measure CDT are capillary zone electrophoresis (CZE) with UV detection as well as HPLC. CE and HPLC methods are preferred over IA, because they provide better separation of the different isoforms [61]. CE methods may be prone to interfering proteins that co-migrate. Of note, interferences of hemoglobin-haptoglobin complexes may occur in HPLC methods coupled with UV detection. Blood–alcohol concentration (BAC). Ethanol is an example of a drug for which the Michaelis–Menten pharmacokinetic model applies and the Michaelis constant (k(m)) for Class I ADH is at a BAC of 2–10 mg/100 mL. This means that the enzyme is saturated with substrate after the first few drinks and that zero-order kinetics is adequate to describe the declining phase of the BAC (BAC > 20 mg/100 mL) [17,18]. After drinking on an empty stomach, the elimination rate of ethanol from blood falls within the range of 10–15 mg/100 mL/h. In non-fasted subjects, the rate of elimination tends to be in the range of 15–20 mg/100 mL/h. In alcoholics during detoxification, because activity of microsomal enzyme (CYP2E1) is boosted, the ethanol elimination rate might be 25–35 mg/100 mL/h. 

The slope of the BAC declining phase is slightly steeper in women compared with men. The gender differences is due to liver weight in relation to lean body mass. It suggests that the physiological range of ethanol elimination rates from blood is from 10 to 35 mg/100 mL/h. In moderate drinkers, it is 15 mg/100 mL/h. However, 19 mg/100 mL/h is more appropriate in binge drinkers or alcoholics. 

## 3. Liver—Coronavirus Disease 2019—COVID-19—A Review and Clinical Approach

### 3.1. Coronavirus Disease 2019

The novel COVID-19 has resulted in a worldwide pandemic. COVID-19 is a ribonucleic acid (RNA) virus which binds to the angiotensin-converting enzyme 2 (ACE-2) and is endocytosed into the host cells [62]. The ACE-2 receptor is found in many organs including the lungs, liver, heart and kidneys [63]. 

Liver enzyme abnormalities were reported early from China in more than 40% of cases [64,65]. An early report of 2273 patients who tested positive for COVID-19 compared to 1108 patients who were negative, showed that positive patients had higher initial and peak ALT levels (28 vs. 21 U/L and 45 vs. 25 U/L, *p* < 0.001) [66]. 

The patients with severe acute liver injury (ALI) were more likely to be admitted to intensive care, require intubation or renal replacement therapy and had higher mortality. They found that the peak ALT level was significantly associated with the outcomes of death or discharge to a hospice (OR 1.14 *p* < 0.044) [66]. Another study showed an increase during the first 2 weeks of hospitalisation in ALT, AST, total bilirubin and GGT [67]. Most patients have mild abnormalities, but a minority have a greater elevation. In one large study, 45% of patients with elevated ALT levels had mild ALI, <2 times the upper limit of normal (ULN), 21% had moderate injury (2–5 times ULN) and 6.4% had severe injury (>5 times ULN) [67]. The significance of the liver function test abnormalities has been debated. A study of 147 hospitalized COVID-19 patients, comparing 93 patients with liver injury to 54 patients without liver injury, found a similar mortality rate [68]. A larger study of 5771 patients with COVID-19 in China found a strong association between elevated AST and mortality with an odds ratio (OR) 4.81 *p* < 0.001 with AST 40–120 U/L and OR 14.87 *p* < 0.001 with AST > 120 U/L [69].

### 3.2. Mechanisms of Liver Function Test Abnormalities

Approximately 3% of patients with COVID have chronic liver disease [70]. Patients with pre-existing liver disease demonstrated a higher proportion (44–81%) of increased liver enzymes at admission [67,71]. GGT is often elevated in non-alcoholic fatty liver disease (NAFLD) patients and there is an increase in GGT in more than half of COVID-19 cases with elevated liver enzymes on admission [67].

Abnormal liver function tests may, however, be due to a non-hepatic cause. Severely ill COVID-19 patients often have high levels of creatine kinase, lactate dehydrogenase or myoglobin due to myositis [72].

AST levels are more frequently elevated as compared with ALT levels in COVID-19 [73,74,75]. Since ACE-2 receptors are predominantly expressed in the cholangiocytes and not the hepatocyte [76], COVID-19 binding probably does not cause direct hepatocyte damage. An autopsy study of 17 patients with COVID-19 found steatosis, lobular and portal tract inflammation, ischemic necrosis, zone 3 hemorrhage or necrosis and platelet—fibrin thrombi in the sinusoids and central vein [77].

The mechanism of liver injury resulting from COVID-19 infection is not clear. It has been proposed that hepatic Kupffer cells mount an inflammatory response which could lead to liver injury [78].

Cardiac failure in severe cases may result in reduced hepatic arterial perfusion and hypoxic hepatitis. Mechanical ventilation with a high positive end expiratory pressure increases right atrial pressure, decreasing venous return resulting in hepatic congestion [72]. The autopsy findings of 32 patients with COVID-19 found that 28 patients had mild cardiomegaly. In addition, 24 patients had histological evidence of myocyte hypertrophy and 20 patients had interstitial fibrosis [79]. 

Many of the drugs used to treat COVID-19 patients have hepatotoxic potential. Patients receiving lopinavir-ritonavir were more likely to develop liver function test abnormalities during admission [80]. 

Remdesivir causes an elevated ALT level in 7% of patients [81]. 

### 3.3. The Effect of COVID-19 Infection on Patients with Pre-Existing Liver Disease

Metabolic-associated fatty liver disease (MAFLD), chronic HCV and chronic HBV are the most common liver disease worldwide. In view of the scale of the COVID-19 pandemic, there will be many cases of infection in patients with underlying liver disease and cirrhosis.

#### 3.3.1. MAFLD

MAFLD is the hepatic manifestation of the metabolic syndrome. Since the risk factors for development of MAFLD (obesity, diabetes mellitus, hypertension) are also risk factors for a worse outcome of COVID-19 infection, there is much interest in determining the effect of COVID-19 infection on such patients [82].

A study of 202 patients in COVID-19 hospitals in China with NAFLD found liver injury in 101 (50%) on admission and 152 (75%) during the period of hospitalization [83,84]. The 39 (19.3%) patients with progressive disease were older, had a higher body mass index (BMI) and a higher percentage of comorbidity and MAFLD. Male gender, age over 60 years, a higher BMI and MAFLD were associated with a higher risk of COVID-19 progression. The patients with MAFLD were also more likely to have abnormal liver enzymes from admission to discharge. In contrast, a study of 589 patients from Qatar found that MAFLD was not an independent predictor of increased mortality, disease severity at presentation or disease progression [84]. It was, however, a predictor for the development of mild-to-moderate liver injury. A further study of 327 patients from China found an increased risk for COVID-19 progression only in patients less than 60 years of age with MAFLD [85].

#### 3.3.2. Hepatitis

Despite chronic HBV hepatitis being common in the Far East, coinfection of HBV and COVID-19 is uncommon. Of 2054 hospitalized cases in China, only 28 (1.36%) had HBV coinfection [86]. A study of 20 patients with COVID-19 and HBV hepatitis found reactivation in 3 of the 19, while 15 had no significant change in the HBV viremia and 2 maintained high HBV DNA levels throughout the admission [87]. 

Steroid therapy, especially dexamethasone, is administered to moderate and severely affected COVID-19 patients. This is unlikely to be a cause of HBV reactivation because of the short period of administration.

The authors suggest that HBV infection may decrease the chance of clinically severe infection. This may be due to “immune exhaustion”, whereby T lymphocytes have a decrease in their ability to produce cytokines as a result of a continuous but inefficient immune reaction to the virus [88].

There are limited data in the literature regarding HCV and COVID-19. A study from Italy of 332 hospitalized COVID-19 patients found 3% with HCV seropositivity; only one of these 10 patients had HCV RNA viremia and none were taking anti-viral medication for HCV. This group was compared to 1527 patients who had treatment for HCV. Of these, 3 (0.196%) aged between 45 to 65 had COVID-19 infection [89]. This again may be due to immune exhaustion.

Autoimmune hepatitis (AIH) often requires prolonged immunosuppressive therapy but the effect on COVID-19 is not clear. A study containing data on 70 patients with autoimmune hepatitis found no differences in the rate of major outcomes between patients with AIH and non-AIH chronic liver disease. Death in the AIH cohort was linked to higher age, higher Child–Turcotte–Pugh score, but not immunosuppression. Propensity score analysis of AIH patients as compared to chronic liver disease patients found no increase in risk of adverse reactions, including death [90]. This supports continuing current immunosuppressive regimens. 

#### 3.3.3. Cirrhosis and Hepatocellular Carcinoma

Cirrhosis as a chronic condition is thought to represent a significant high-risk comorbid condition for a worse outcome of COVID-19 infection. Initial reports of a mortality rate approaching 30–40% in those patients with cirrhosis but the major causes of death were pulmonary causes [91]. The death rate increased in parallel to the severity of the cirrhosis. There was, however, no control group of patients with COVID-19 without cirrhosis. Bajaj et al. have reported their results of a multi-centre study of 37 patients with cirrhosis and COVID-19 as compared to 108 patients with COVID-19 and 127 patients with cirrhosis [92]. There was a higher mortality in the COVID-19 infected cirrhosis patients compared to patients with COVID-19 alone (30% vs. 13%, *p* = 0.03). There was, however, no significant difference between patients with cirrhosis and COVID-19 and patients with just cirrhosis (30% vs. 20% *p* = 0.06).

In addition, a multi-centre North American Study found that cirrhosis alone has an adverse effect on death and 90-day readmissions, that is greater than COVID-19 alone. In addition, those cirrhosis patients with COVID-19 were more likely to be readmitted for events not connected to liver disease [93].

#### 3.3.4. Alcoholic Liver Disease

The group of patients with alcoholic liver disease may be amongst the most severely affected by COVID-19 and have a higher risk of infection [94]. Reasons for this include the presence of comorbidities such as obesity, the metabolic syndrome and smoking, an increased risk of infections due to a weakened immune system, logistic difficulties in maintaining ambulatory appointments, psychological decompensation and increased consumption of alcohol or relapse of alcoholism due to social isolation [95].

#### 3.3.5. Liver Transplant

The issue of liver transplantation in the era of the COVID-19 pandemic is complex due to the risk to both the donor and the recipient. There are risks from delayed evaluation for transplant and risks while undergoing evaluation. Telemedicine is used to reduce visits to transplant centers.

Recently, two studies describing the experience of treating liver transplant patients with COVID-19 have been published. A study from 35 healthcare organizations in the USA found a higher risk of hospitalization (RR1.72, *p* = 0.043) in the liver transplant patients. There was, however, no difference in the risk of mortality, thrombosis, or the need for intensive care admission [96].

Another report from Spain included 111 liver transplant patients who were diagnosed with COVID-19 [97]. After a mean 23 days of follow-up, 96 individuals (86.5%) required hospitalization and 22 (19.8%) of these received respiratory support. However, the mortality rate of 18% was less than the age- and gender-matched control group in the Spanish Liver Transplant Registry. In this study, baseline treatment with mycophenolate, but not other immunosuppressive, was found to be associated with a worse outcome.

### 3.4. Kaplan Medical Center Experience with COVID-19

At Kaplan Medical Center, in Israel, between March and August 2020, we treated 175 patients hospitalized with COVID-19: 93 (53%) were male (average age 65.2 years) and 82 (47%) were female (average age 64.3 years). 

The severity of the disease was determined by: the presence of fever, cough, weakness, loss of taste and smell (mild symptoms of COVID-19); clinical or X-ray diagnosis of COVID-19 pneumonia (moderate) and severe. The severe COVID-19 diagnosis presented one of the following criteria: respiratory rate of over 30 per minute; blood oxygen saturation of 93% or lower without oxygen support and or PaO_2_/FiO_2_ ratio lower than 300.

In addition, a history of pre-existing liver disease was noted when present.

On admission, 83 patients were in mild condition, 37 moderate and 53 severe. At discharge, 106 patients were mild, 45 moderate and 6 were in a severe condition. Sixteen patients died in hospital. 

Prior to admission, seventeen patients (9%) had ultrasound evidence of liver disease, two patients (1%) had chronic HBV hepatitis, two (1%) had chronic HCV hepatitis and one patient presented with autoimmune hepatitis. There was a statistically significant relationship between higher admission AST levels and severity of admission COVID-19 infection condition on admission [98,99].

There was no statistical relationship between treatment with remdesivir and change in liver enzymes during admission (*p* = 0.797, 0.799, 0.495, 0.942). We found a statistically significant relationship between underlying liver disease and COVID-19 severity on discharge (*p* = 0.039). 

### 3.5. Suggested Clinical Approach

At our medical center, we were early adopters of the use of convalescent plasma. The initial reports of a lack of clinical efficacy were, in our view, related to the delay in administration [99]. The evidence from the Mayo Clinic is clear that early administration of convalescent plasma with high concentration of antibodies against COVID-19 is effective. 

We suggest that patients admitted for COVID-19 should have their liver enzymes checked on admission. For those patients who are in the early stages of their illness and are moderately to severely ill, intravenous convalescent plasma (or monoclonal antibodies if available) should be considered. The status regarding common chronic liver diseases (MAFLD, HCV and HBV hepatitis) may be determined from the electronic medical record. 

Those patients with chronic HBV hepatitis should continue on their nucleoside analogue therapy and direct-acting anti-virals (DAA) for HCV hepatitis should also continue. For patients with MAFLD, monitoring and addressing risk factors is required. For autoimmune hepatitis, we suggest trying to keep the steroid dose less than 20 mg prednisone per day and to avoid stopping azathioprine or mycophenolate unless there is a profound lymphopenia. 

**Figure 5:**
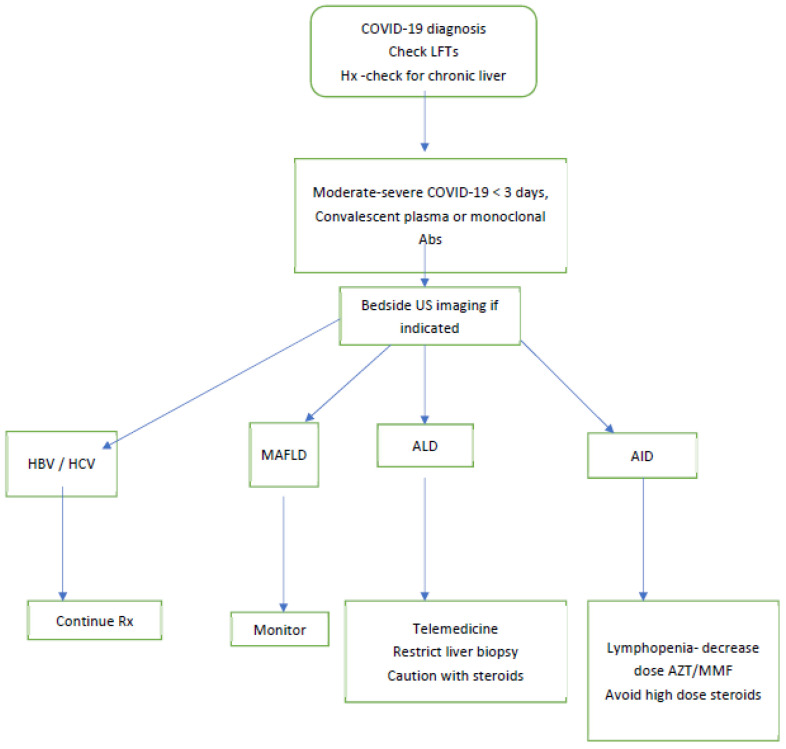
Proposed clinical therapy and monitoring of patients during hospitalization. Proposed clinical therapy and monitoring of patients during hospitalization.

Figure 5 illustrates our approach.

The pandemic related to COVID-19 has implications for patients with both acute and chronic liver disease. Patients with chronic liver disease should be vaccinated against COVID-19 as soon as it becomes available. A considered approach, as detailed above, should be adopted for patients with liver enzyme disturbance and COVID-19, patients who are liver transplant candidates, or have suspected HCC with concurrent COVID-19.

## 4. Personalized, Precision Medicine and Non-Invasive Biomarkers to Determine Severity of Alcohol-Induced Hepatocytotoxicity in Human Alcoholic and Non-Alcoholic Liver Disease

### 4.1. Non-Invasive Biomarkers

The recent development of non-invasive tools to diagnose various disease stages of liver disease by elastography or room temperature susceptometry has significantly improved the screening of patients with liver diseases [100].

The chronic injury of the liver due to alcohol or drug-induced hepatocytotoxicity of the disease and to monitor the evolution of fibrosis in patients using molecular markers in urine [101] is required. The Bannaga team recruited, in a multicentre combined cross-sectional and prospective diagnostic test validation study, 129 patients with varying degrees of liver fibrosis and 223 controls without liver fibrosis. Urinary low molecular weight proteome was analysed by capillary electrophoresis coupled to mass spectrometry [101]. The sequence-identified peptides were fragments of collagen chains, uromodulin and Na/K-transporting ATPase subunit γ. Moreover, the researchers identified putative proteolytic cleavage sites, e.g., eight were specific for matrix metallopeptidases and two for cathepsins [101].

Liver damage due to high alcohol consumption produces a cytokine storm syndrome characterized by the release of pro-inflammatory cytokines. The activation of the immune system is a host defense mechanism [102,103,104,105,106]. In response to chronic, heavy alcohol exposure, hepatocytes express and secrete chemokines [107,108,109,110]. 

The role of inflammation in chronic liver disease may lead to acute-on-chronic liver failure (ACLF) [109]. ACLF syndrome includes high inflammation due to the presence of bacterial products such as pathogen-associated molecular patterns (PAMPs), and virulence factors such as acute viral infection. Pathogen-associated molecular patterns elicit inflammation via innate pattern-recognition receptors (PRRs), whereas virulence factors generally trigger inflammation via functional feature identification. Endogenous inducers are called danger-associated molecular patterns (DAMPs) and include molecules released by necrotic cells and products of extracellular matrix breakdown [105,107,108,109].

### 4.2. Apoptosis

Increasing evidence suggests an important role for hepatocyte apoptosis in the progression of ALD [109,110]. Several other forms of cell death have been described, including necrosis, necroptosis, pyroptosis autophagic cell death, and others [111,112,113,114]. Both apoptosis and necrosis have also been proposed to be responsible for the development and progression of liver fibrosis [114]. Early during apoptosis, caspases are activated and cleave various substrates including cytokeratin 18 (K18) [113,114]. K18 is a member of the intermediate filament family of cytoskeletal proteins [115]. Cytokeratine-generated cleavage fragments of K18 can be detected in serum by the M30 antibody, which specifically labels early apoptotic fragments of cells [113,114,115,116,117,118,119,120,121,122]. An increase of apoptotic activity has been demonstrated in heavy drinkers undergoing alcohol detoxification [113]. The data from our detoxification patients suggested that alcohol may inhibit apoptosis and therefore diminish an important clearance pathway of tumor cells [113]. Our study in familial NAFLD indicated a link between reduced apoptotic activity and incidence of HCC [122].

### 4.3. Alcohol Misuse and Cancer

In a previous study, we described the link between alcohol misuse and rare cancer [123]. The cytokines that regulate angiogenesis are the subject of intense interest in clinical setting. 

The characterization of the vascular endothelial growth factor (VEGF) and its receptors leads to new therapeutic anti-angiogenesis. This is an emerging approach for the treatment of ischaemic heart disease, as well as rare diseases such as POEMS. 

Polyneuropathy, organomegaly, endocrinopathy, M-protein, skin changes (POEMS) syndrome comprise a paraneoplastic syndrome due to an underlying plasma cell neoplasm. The major criteria for the syndrome are polyradiculoneuropathy, clonal plasma cell disorder, sclerotic bone lesions, elevated vascular endothelial growth factor, and the presence of Castleman disease. Minor features include organomegaly, endocrinopathy, characteristic skin changes, papilledema, extravascular volume overload, and thrombocytosis. The syndrome is rare and can be mistaken for other neurologic disorders, such as chronic inflammatory demyelinating poly-radiculo-neuropathy. We showed that, by monitoring the levels of VEGF in POEM patients, we monitor the severity of the disease, its possible progression and the effect of the therapeutic intervention [123].

Finally, we describe the discovery of a novel class of intracellular proteins (suppressors of cytokine signaling, SOCS) that may play a key role in the downregulation of cytokine signaling. As key regulators of cytokine signaling, these molecules represent an exciting new target for drug discovery. Previously, we showed that transforming growth factor-β (TGFβ) is crucial for liver fibrogenesis [124]. The inhibition of TGFβ signalling in hepatic stellate cells (HSCs) can effectively inhibit liver fibrosis. microRNAs (miRNAs) have emerged as key regulators in modulating TGFβ signaling and liver fibrogenesis. The liver is maintaining a balance between muscle growth and degradation. The mTOR signalling pathway increases protein synthesis via phosphokinase B. This pathway is increased by physical exercise, testosterone, insulin and insulin-like growth factor 1(IGF-1). Also of note are the proliferation and activation of the satellite cells [125,126]. 

Ubiquitin-dependent proteasomal degradation is the major pathway involved in muscle degradation, and the second is autophagy. Increased level of inflammation leads to increased muscle degradation and overproduction of pro-inflammatory cytokines like adiponectin, interleukin (IL)-6 and TNF-alpha, which have fibrogenic and oxidative effects. Autophagy levels are higher in cirrhotics, and alcohol can stimulate the autophagy pathway. Reduced physical activity also leads to reduction in the release of myokines, which normally help to maintain muscle mass [124,125]. An important factor in alcoholic liver disease is nutrition. A study from the United States, which included veterans, reported higher mortality (>80%) among alcoholic hepatitis patients with total calorie intake less than 1000 kcal/d, compared with those consuming >3000 kcal/d. The risk of mortality was inverse proportional with the daily calorie consumption. In a control trial, 6 months of survival was shown to be similar among patients who received intensive enteral nutrition plus methyl-prednisolone compared with those who received conventional nutrition plus methyl-prednisolone. Patients who consumed daily calories <21.5 kcal/kg/d died in higher proportion when compared with the individuals who consumed more calories (65.8% vs. 33.1%, *p* < 0.001) [127]. 

## 5. Conclusions

Alcohol-induced liver injury has multiple problems. The present review has the intention to raise awareness of the importance of understanding the scientific and medical up-to-date knowledge in the field. 

Assessing and treating the severity of alcoholic liver injury and non-alcoholic steatohepatitis, as well as the different aspects including viral infection, malnutrition and other co-morbidities, are essential components of treating patients with liver injury. Nutritional assessment and management can be improved by close cooperation between attending physicians, hepatologists, laboratory medicine specialists and dieticians. Involvement of patients and their families will ensure improvement in educational training regarding medical and nutritional needs and will lead to associated benefits to the patients.

## Figures and Tables

**Table 1:** ROS and selected potentially toxic intermediates.

Acetaldehyde C_2_H_4_O	Singlet radical ^1^O_2_	Alkoxyl radical RO**^.^**
Ethoxy radical CH_3_CH_2_OS	Superoxide radical HO**^.^**_2_	Peroxyl radical ROO**^•^**
Hydroxyethyl radical CH_3_C**^.^**HOH	Hydrogen peroxide H_2_O_2_	Lipidperoxides
Acetyl radical CH_3_CHO	Hydroxyl radical HO**^•^**	

Adapted from a table published in an open-access journal [13].

**Table 2:** Selected substrates of the hepatic microsomal CYP 2E1.

Acetaldehyde	Chloroform	1,2-Dibromoethane	Methyl t-butyl ether
Sevoflurane			
Acetol	1-Chloropropane	Diethylether	Methoxyflurane
Styrene			
Acetone	Chlorzoxazone	Dimethylformamide	Monochlorobenzene
1,1,1,2-Tetrachloroethane			
Acetaminophen	1,1-Dichloroethane	Enflurane	4-Nitrophenol
1,1,2,2-Tetrachloroethane			
Aniline	1,2-Dichloroethane	Ethanol	Nitrosamines
Tetrachloroethylene			
Benzene	1,1-Dichloroethylene	Ethylbenzene	
N-nitrosodimethylamine	Trichloroethylene		
Bromobenzene	*cis*-1,2-Dichloroethylene	Halothane	*n*- Pentane
Toluol			
n- Butanol	*trans*-1,2-Dichloroethylene	*n*-Hexane	Phenol
1,1,1-Trichloroethane			
Caffeine	Dichloromethane	Isoflurane	n-Propanol
1,1,2-Trichloroethane			
Carbon tetrachloride	1,2-Dichloropropane	Methanol	Propylbenzene
Vinylchloride			

Preferred substrates of cytochrome P450 2E1. The table is derived from an open-access article containing references for each chemical [13].

## Abbreviations

ACE-2	angiotensin-converting enzyme 2
AH	alcoholic hepatitis
AIH	autoimmune hepatitis
ALD	alcohol-related liver disease
ALI	acute liver injury
ALT	alanine aminotransferase (glutamic-pyruvic transaminase, GPT)
ALP	alkaline phosphatase
AMA	anti-mitochondrial antibody
ANA	antinuclear antibody
AST	aspartate aminotransferase (glutamic-oxalic- transaminase, GOT)
ASH	alcoholic steatohepatitis
BMI	body mass index
CCK	caspase cleaved cytokeratin (8 & 18)- M30-M 65
COVID	Corona virus disease
DAA	direct-acting anti-virals
DAMPs	danger-associated molecular patterns
DNA	deoxyribonucleic acid
EGF	epithelial growth factor
ER	endoplasmic reticulum
EtG	ethanol glucuronide
EtS	ethanol sulphate
FDA	Food and Drug Administration
GGT	γ-glutamyl-transferase
GSH	glutathione
HBV	hepatitis virus B
HCV	hepatitis virus C
HER	hydroxyethyl radical
HGF	hepatocyte growth factor
HSC	hepatic stellate cells
IL	Interleukin: IL-6, IL-8
INR	International Normalized Ratio
KC	Kupffer cells
LPS	lypo-poli-saccharides
MAA	malon-dialdehyde
MAFLD	metabolic associated fatty liver disease,
Mean ± SD	mean ± standard deviation
MEOS	microsomal ethanol oxidizing system
NAD^+^	nicotinamide adenine dinucleotide,
NADH	reduced nicotinamide adenine dinucleotide
NADP^+^	nicotinamide adenine dinucleotide phosphate,
NADPH	reduced nicotinamide adenine dinucleotide phosphate,
PAMPs	pathogen-associated molecular patterns
PhET	phosphatidyl ethanol
POEMS	Polyneuropathy, organomegaly, endocrinopathy, M-protein, skin changes syndrome
PPAR alpha	peroxisome proliferator—activated receptor alpha,
PRRs	pattern-recognition receptors
RBC	red blood cell
RNA	ribonucleic acid
ROS	reactive oxygen species
SOCS	suppressor of cytokine signaling
SREBP-1	sterol regulatory element binding protein-1
TGF-β	transforming growth factor beta
TNF-alpha	Tumor necrosis factor alpha
TLR4	toll-like receptor 4
U/L	Unit/Liter
ULN	Upper the limit of normal
VEGF	vascular endothelial growth factor
